# Video Assessment of Surgical Performance in Robotic Gastroenterostomy During Pancreatoduodenectomy: Evaluating the Learning Curve and Risk of Delayed Gastric Emptying

**DOI:** 10.1097/SLA.0000000000006692

**Published:** 2025-03-12

**Authors:** Maurice J.W. Zwart, Bram L.J. van den Broek, Diederik S.J. Paijens, Sabrina L.M. Zwetsloot, Annalisa Comandatore, Olivier R. Busch, Khé T.C. Tran, Misha D. Luyer, Jennifer Schreinemakers, Jan H. Wijsman, George P. van der Schelling, Ignace H.J.T. de Hingh, Jan S.D. Mieog, Bert A. Bonsing, Kosei Takagi, Roeland F. de Wilde, Luca Morelli, Herbert J. Zeh, Amer H. Zureikat, Melissa E. Hogg, Bas Groot Koerkamp, Marc G. Besselink

**Affiliations:** * Department of Surgery, Amsterdam UMC, University of Amsterdam, Amsterdam, The Netherlands; † Department of Surgery, Cancer Center Amsterdam, Amsterdam UMC, The Netherlands; ‡ Department of Surgery, Erasmus University Medical Center, Rotterdam, The Netherlands; § Department of General Surgery and Department of Translational Research and New Technologies in Medicine and Surgery, University of Pisa, Pisa, Italy; ∥ Department of Surgery, Catharina Medical Center, Eindhoven, The Netherlands; ¶ Department of Surgery, Amphia Ziekenhuis, Breda, The Netherlands; # Department of Surgery, Leiden University Medical Center, Leiden, The Netherlands; ** Department of Surgery, University of Texas Southwestern Medical Center, Dallas, TX; †† Department of Surgery, Division of GI Surgical Oncology, University of Pittsburgh Medical Center, Pittsburgh, PA; ‡‡ Department of Surgery, NorthShore University HealthSystem, Evanston, IL

**Keywords:** DGE, gastrojejunostomy, OSATS, pancreatoduodenectomy, robotic

## Abstract

**Objective:**

To identify learning curves for robotic gastroenterostomy (RGE) during robotic pancreatoduodenectomy (RPD) and the predictive value of the objective structured assessment of technical skills (OSATS) score for delayed gastric emptying (DGE) according to the Birkmeyer and colleagues and University of Pittsburgh Medical Center method.

**Background:**

In some series, RPD has been associated with an increased risk of DGE. It is unclear whether this is attributable to learning curve. Improved surgical performance and experience have not yet been linked to a decrease in DGE in RPD.

**Methods:**

Post hoc study of the prospective multicenter (LAELAPS-3) training program, including videos of RGE during RPD. Surgical performance was scored with OSATS by 2 blinded graders. The main outcomes are the combined OSATS scores of 2 blinded graders over time (learning curve). Secondary outcome is the correlation between OSATS scores and clinically relevant DGE (grade B/C).

**Results:**

Videos from 192 RGE anastomoses were included. DGE occurred in 42/192 (21.9%) patients. The mean OSATS score was 22.4 (SD ± 5.1) and predicted DGE Area under the curve (AUC): 0.668, *P* < 0.001). The predictive OSATS elements for DGE were gentleness (AUC: 0.719, *P* < 0.001), instrument handling (AUC: 0.595 *P* = 0.043), tissue exposure (AUC: 0.625, *P* = 0.009), and summary score (AUC: 0.665, *P* < 0.001). An OSATS score >25 was associated with a 59.9% reduced relative risk of grade B/C DGE [11.3% (8/71) vs 28.1% (34/121); odds ratio (OR): 0.325, *P* = 0.006]. Cumulative sum analysis of RGE-OSATS identified a turning point at 34 procedures [25.7% (36/140) before vs 11.5% (6/52) after; OR: 0.156, *P* = 0.035]. On multivariable analysis for grade B/C DGE, OSATS ≤25 remained an independent risk factor (OR: 2.907, *P* = 0.028).

**Conclusions:**

Better surgical performance during gastroenteric anastomosis in RPD, as assessed by OSATS, is associated with a reduced rate of grade B/C DGE. OSATS could serve as a tool for competency-based training programs and quality-controlled implementation of RPD.

Pancreatoduodenectomy (PD) is one of the most complex gastrointestinal operations. While open PD is the standard, a minimally invasive approach has become more frequently used in some high-volume centers.^1–3^ In trained and experienced centers, robotic PD (RPD) has proven to be as safe and efficient as open PD, with benefits in terms of overall length of hospital stay, complication rates, and wound infection rates.^4–10^ However, postoperative morbidity remains substantial.^5,7,11^ Especially the rate of delayed gastric emptying (DGE) seems to be increased after RPD, occurring in 14% to 50% of patients.^3,12–16^ This substantially contributes to a prolonged hospital stay (+5.5 days) and higher readmission rates (+79%).^1,4,17^

A recent study showed that certain technical aspects of the gastrojejunostomy reduce the risk of DGE, for example, the angulation of the efferent and the afferent loop.^11^ Besides technical factors, improved surgical performance is known to reduce morbidity and mortality, for example, pancreatic fistula after RPD.^18,19^ Birkmeyer et al^19^ used the objective structured assessment of technical skills (OSATS)^20^ to assess laparoscopic surgical performance and showed that high OSATS scores were associated with lower complication rates. Lower OSATS scores, in contrast, were associated with higher complication rates.

While RPD allows for high-quality video recording, analysis of RPD videos using OSATS gives the opportunity to monitor the performance of an individual surgeon.^18,21^ Kendrick et al^22^ emphasized that OSATS scores could objectify the number of RPD procedures that individual surgeons need to perform to complete the learning curve. However, the effect of surgical performance of robotic gastroenterostomy (RGE) on the rate of DGE has not yet been assessed in RPD.^23^

The study aimed to identify learning curves and assess the impact of the surgical performance of RGE as assessed by OSATS score on the rate of DGE grade B/C after RPD.

## METHODS

### Study Design

This is a post hoc study of the multicenter prospective LAELAPS-3 cohort study. The study was approved by the Institutional Review Board of Amsterdam UMC (under study number: #W17_149) and subsequently by all participating centers.

### Setting and Participants

All RPD procedures performed between January 2017 and December 2021 from 5 Dutch hospitals were included (Amphia Hospital Breda, Amsterdam UMC, Catharina Hospital Eindhoven, Erasmus MC, and Leiden University Medical Center), being 3 university medical centers and 2 teaching hospitals. All indications for RPD were included. Patients were excluded when a full-length video of the gastric anastomosis was unavailable. Full length of the gastric anastomosis is defined as starting setting up of the jejunal loop to finishing the last stitch of the anastomosis, including any revision during the same procedure.

### Variables

Baseline variables included: age, body mass index, sex, gastroenterologic comorbidity, diabetic comorbidity, adenocarcinoma, and neoadjuvant therapy. Operative technique variables included: stapled versus hand-sewn anastomosis technique, suturing time (including the enterotomy and, if applicable, the stapling time), type of suture material used, angle type classification of gastrojejunostomy according to Masui et al,^24^ and modified by Jung et al^11^ (Appendix 1, Supplemental Digital Content 1, http://links.lww.com/SLA/F432), cautery transection time (defined as the time that energy was used to perform the enterotomy), and consecutive procedure number by surgeon. Surgeon characteristic variables included: employed at a university medical center, whether the surgeon was an attending, the years of surgical experience, the years of minimally invasive surgical experience, whether the surgeons’ annual volume exceeded 20 PDs (open and minimally invasive) per year, and prior experience in laparoscopic PD.

Surgical performance was graded by 2 blinded graders (M.J.W.Z. and B.L.J.v.d.B.) using the OSATS score.^9,10^ The OSATS score consists of 6 variables:

- Gentleness: Gentle tissue handling that does not result in injury.
- Time and motion: Economy of motion, maximum efficiency.
- Instrument handling: Fluid use of instruments without awkwardness.
- Flow of operation: Smooth transitions from one part of the operation to another.
- Tissue exposure: Retraction that allows for good visualization and proper tissue alignment.
- Overall technical skill: Overall assessment of technical skill.

**FIGURE 1:**
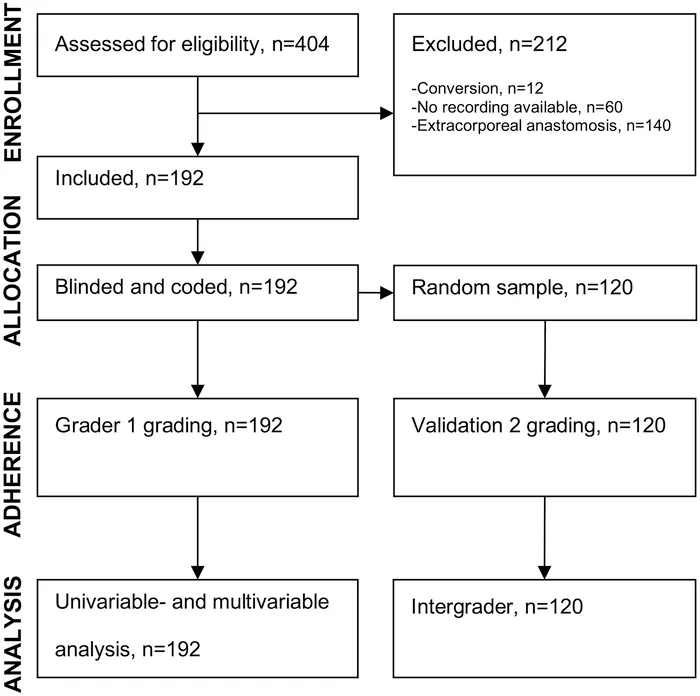
Flowchart of inclusion.

All components are graded between 1 and 5, with a score of 1 being deficient/traumatic and 5 being flawless. When adding the scores from all components of OSATS, an OSATS score for surgical performance is calculated. The grader analyzed RGE videos independently and was blinded to patient, outcome, participating center, and operating surgeon. Blinding was obtained by anonymizing the videos with a random 4-digit code generated in SPSS. A third grader, a surgical research fellow in hepato-pancreato-biliary surgery (A.C.), scored independently as a validation grader. A random sample of 120 procedures was generated using a computer. See Figure 1 for more details.

### Outcomes

The primary outcome was a single surgeon learning curve identified with OSATS. Secondary outcome was the predictive value of OSATS for postoperative DGE. DGE was defined as DGE in the absence of mechanical obstruction and graded according to the International Study Group of Pancreatic Surgery (Table 1).

**TABLE 1:** Definition of DGE According to the ISGPS

ISGPS DGE	Nasogastric tube needed	Unable to tolerate solid diet
Grade A	4–7 d or reinsertion after POD 3	By POD 7
Grade B	8–14 d or reinsertion after POD 7	By POD 14
Grade C	>14 d or reinsertion after POD 14	By POD 21

ISGPS indicates International Study Group of Pancreatic Surgery; POD indicates postoperative day.

### Gastrojejunostomy Technique

The procedure was performed as described by the LAELAPS-3 proctors, including the transection of the stomach and the pylorus-resecting nature.^11^ Based on surgeon preferences from open surgery, minor alterations to the standard RPD technique were allowed. The transection of the stomach corpus/stomach antrum or duodenal bulb was performed by stapler. In most patients, the length of the bowel between the hepaticojejunostomy and gastric anastomosis was measured at 50 to 60 cm. These enterostomies were performed using a standard antecolic, isoperistaltic technique (no Roux-en-Y reconstruction nor Braun enterostomies).

### Statistical Methods

Data were analyzed using IBM SPSS statistics for Windows version 28 (IBM Corp). Normally distributed continuous data are presented as mean and SD. Non-normally distributed continuous data are presented as median and interquartile range (IQR) or 95% CI. Categorical (binary, nominal, and ordinal) data are presented as frequencies and percentages. Outcomes are compared and assessed for significance (2-tailed *P* value of <0.05) with the Student *t* test for normally distributed data, χ^2^ test for frequencies in one or more categories, and Mann-Whitney *U* test for non-normally distributed data.

The OSATS score was categorized into 4 groups by quartiles (quartile 1, quartile 2, quartile 3, and quartile 4). Logistic regression was used to test the predictability of the OSATS. Likert-Scale ordinal data were also presented in means and SDs, as this allows more insight into the effect size.^25^ For the intragrader and intergrader correlation coefficient analysis, the Spearman rho expressed as 95% CIs. In accordance to Müller et al,^23^ learning curve analyses were performed to investigate the association of the OSATS score and the consecutive case number of each surgeon. Cumulative sum analysis was used to determine the learning curve turning point for the OSATS score. The turning point was defined as the moment when the learning started to decrease in slope angle. The cutoff analysis was performed with logistic regression, to identify an OSATS score to be reached to minimize the risk of DGE.

Sensitivity analysis for concomitant complications that could cause DGE was performed through subgroup analysis for postoperative complications comprising: pancreatic fistula, postoperative pancreatic hemorrhage, and bile leakage. A multivariable analysis was performed, including patient, surgeon, and anastomosis characteristics that had a *P* value <0.100 were included in a univariable analysis for grade B/C DGE. Finally, a sensitivity analysis was performed, distilling the most impactful elements of the OSATS score and determining their combined value.

## RESULTS

### Eligibility

Overall, 404 RPDs were included from the 5 Dutch centers. A total of 192 patients were finally included after the exclusion of conversion to open surgery in 12 patients (3.0%), recording not available in 60 (14.9%) patients, and an extracorporeal gastroenterostomy anastomosis in 140 patients (34.7%). For the flowchart of enrollment, see Figure 1. A DGE grade B/C occurred in 42/192 patients (21.9%), of which 19/192 were grade C (9.9%). In extracorporeal gastroenterostomy (including conversions), the rate of DGE rate grade B/C was similar to the intracorporeal gastroenterostomy, 45/152 (29.6%) versus 62/252 (24.6%), *P* = 0.270.

### Patient and Surgeon Characteristics

The mean patient age was 67 years (SD ± 10), median body mass index was 25 kg/m^2^ (IQR: 23–28), 103/192 patients were males (53.6%), and 150/192 (78.1%) patients had at least one or more comorbidity. Most patients were ASA 2, n = 115/192 (59.9%). See Table 2 for more details.

**TABLE 2:** Characteristics of 192 Patients After RPD

Baseline characteristics	Total (n = 192)	OSATS-RGE ≤25 (n = 121)	OSATS-RGE >25 (n = 71)	*P*
Age (yr), median (IQR)	67 (60–74)	68 (61–73)	70 (59–74)	0.064
BMI (kg/m^2^), median (IQR)	25 (23–28)	25 (23–28)	25 (23–27)	0.581
Sex (M)	103 (53.6)	65 (53.7)	38 (53.5)	0.979
ASA physical status ≥3, n (%)	65 (33.9)	40 (33.1)	25 (35.2)	0.761
Gastrointestinal comorbidity, n (%)	62 (32.3)	40 (33.1)	22 (31.0)	0.787
Diabetes mellitus, n (%)	37 (19.3)	23 (19.0)	14 (19.7)	0.904
Adenocarcinoma, n (%)	140 (72.9)	90 (74.4)	50 (70.4)	0.551
Neoadjuvant therapy, n (%)	25 (13.0)	11 (9.1)	14 (19.7)	0.035
Operative technique				
Anastomosis type (stapled), n (%)	75 (39.1)	52 (43.0)	23 (67.8)	0.147
Pylorus resecting PD, n (%)	182 (94.8)	115 (95.0)	67 (94.4)	0.839
Suture material (V-loc use), n (%)	52 (27.1)	36 (29.8)	16 (22.5)	0.277
Angle type (type I/II/III)	120/21/51	77/16/28	43/5/23	0.215
Cautery transection (s)	76 (36–120)	80 (29–127)	72 (40–112)	0.918
RGE time (min), median (IQR)	45 (14–97)	52 (36–65)	47 (32–57)	0.109
Suture time, median (IQR)	28 (18–39)	28 (18–40)	29 (17–36)	0.435
Procedures, consecutive number	22 (10–53)	18 (9–34)	22 (10–53)	0.116
Surgeon characteristic				
University medical center, n (%)	155 (80.7)	97 (80.2)	58 (81.7)	0.945
Attending surgeon, n (%)	119 (62.0)	85 (70.2)	34 (47.9)	0.005
Surgical experience (yr), median (IQR)	15 (5–16)	15 (7–16)	7 (5–16)	0.083
MI surgical experience	2 (1–14)	2 (1–14)	2 (1–14)	0.869
Surgeon annual volume >20 PDs	159 (17.2)	101 (83.5)	58 (81.7)	0.714
Prior experience in LPD	55 (28.6)	33 (27.3)	22 (31.0)	0.453
Postoperative outcomes				
Complications CD grade ≥3, n (%)	75 (39.1)	49 (40.5)	26 (36.6)	0.595
Grade B/C DGE, n (%)	54 (28.1)	34 (28.1)	8 (11.3)	0.006
Grade C only, n (%)	19 (9.9)	13 (10.7)	6 (8.5)	0.607
Grading				
Total OSATS score, mean (95% CI)	22.3 (21.6–23.1)	19.4 (18.7–20.1)	27.5 (27.2–27.9)	<0.001
Gentleness, mean (95% CI)	3.5 (3.3–3.7)	3.0 (2.8–3.2)	4.4 (4.2–4.5)	<0.001
Time and motion, mean (95% CI)	4.0 (3.9–4.2)	3.6 (3.4–3.8)	4.7 (4.6–4.8)	<0.001
Instrument handling, mean (95% CI)	4.0 (3.9–4.2)	3.6 (3.5–3.8)	4.8 (4.7–4.9)	<0.001
Flow of operation, mean (95% CI)	3.7 (3.5–3.9)	3.2 (3.0–3.4)	4.7 (4.5–4.8)	<0.001
Tissue exposure, mean (95% CI)	3.9 (3.7–4.1)	3.3 (3.1–3.6)	4.8 (4.6–4.9)	<0.001
Summary score, mean (95% CI)	3.3 (3.1–3.4)	2.7 (2.5–2.9)	4.3 (4.1–4.4)	<0.001

BMI indicates body mass index; CD, Clavien-Dindo; LPD, laparoscopic pancreatoduodenectomy.

The RGE was performed by 8 surgeons with a median surgical experience of 15 years (IQR: 5–16), of which 2 years (IQR: 1–14) with RPD. Two surgeons reported annual case surgeon volume of RPD <20, and 6 surgeons reported annual case volume of RPD ≥20. Three of the 9 surgeons had prior experience in laparoscopic PD.

### Operative Outcomes

The vast majority of patients (182/192, 94.8%) underwent a pylorus resecting PD. The RGE was stapled in 75/192 patients (39.1%). Stratafix, JNJ ETHICON, Plaza New Brunswick (Barbed suture) was the most frequently used suture material (140/192, 72.9%). The median RGE time was 48 minutes (IQR: 34–61), of which suture time was 28 minutes (IQR: 18–39). Intraoperative revision of the RGE was done in 5/192 (2.7%) procedures. Type I (<30 degrees to vertical) was the most common anastomosis orientation: type I 120/192 (62.5%), type II 21/192 (10.9%, and type III 51/192 (26.6%). The transection time with the use of cauterization was 76 seconds (IQR: 36–120). Mean total OSATS scores were 22.4 (SD ± 5.1), mean separate OSATS element scores were: gentleness 3.5 (SD ± 1.2), time and motion 4.0 (SD ± 1.0), instrument handling 4.1 (SD ± 1.0), tissue exposure 3.9 (SD ± 1.2), flow of operation 3.7 (SD ± 1.1), and summary score 3.3 (SD ± 1.1).

### Objective Structured Assessment of Technical Skills Learning Curve

**FIGURE 2:**
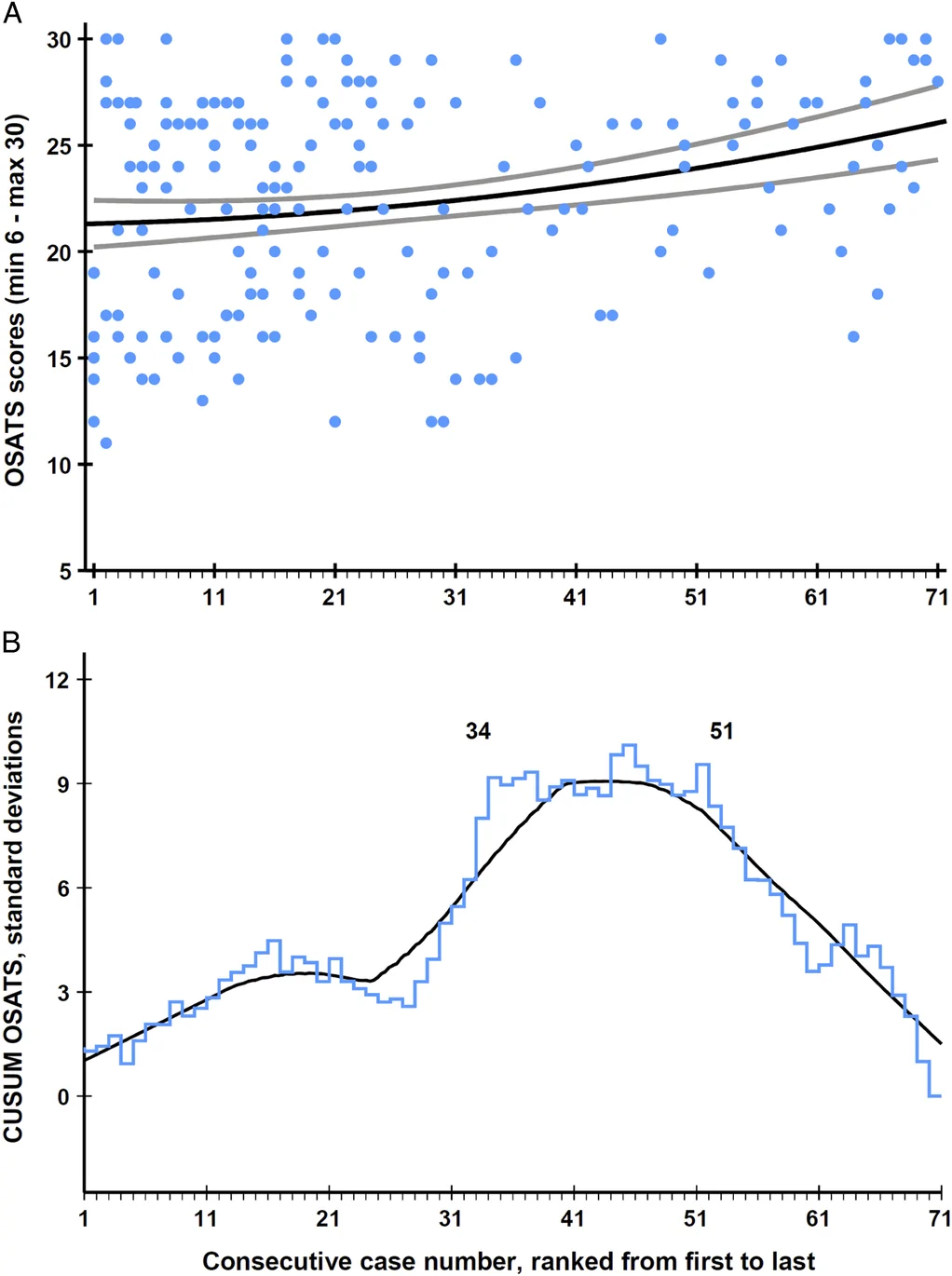
A and B, Distribution of OSATS total scores during the surgeon’s maturation of experience.

The total OSATS score increased significantly along the maturation of experience: Correlation coefficient 0.254, *P* = 0.002. All OSATS elements demonstrated a significant increase along the maturation of the experience. Cumulative sum analysis of the OSATS data over time identified a turning point after 34 RPDs. Hereafter, there was a stable plateau, and after 51 cases, the curve demonstrated a downward slope. For more information, see Figure 2. The rate of grade B/C DGE was higher before the stabilization of the learning curve compared with after the learning curve [25.7% vs 11.5%, *P* = 0.035; OR: 0.377 (95% CI: 0.148–0.956)]. The relative risk reduction was 55.2%. The rate of grade C DGE was not significantly different before and after 34 procedures (10.7% vs 7.7%, *P* = 0.533). The rate of grade B/C DGE was higher before 51 procedures compared with hereafter (24.8% vs 6.5%, *P* = 0.023).

### Objective Structured Assessment of Technical Skills and Delayed Gastric Emptying

**FIGURE 3:**
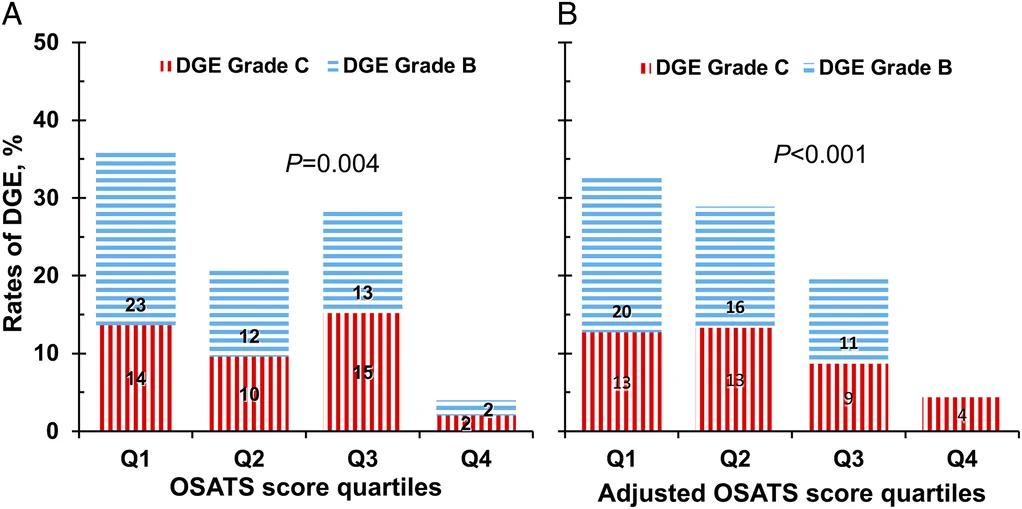
Proportion of DGE after RPD per OSATS quartile. A, Rates of postoperative DGE along quartiles of the OSATS score, incrementally from low (Q1) to high scores (Q4). B, Rates of postoperative DGE along quartiles of the adjusted OSATS score (significant predictive elements: gentleness, instrument handling, tissue exposure, and summary score), incrementally from low (Q1) to high scores (Q4).

**FIGURE 4:**
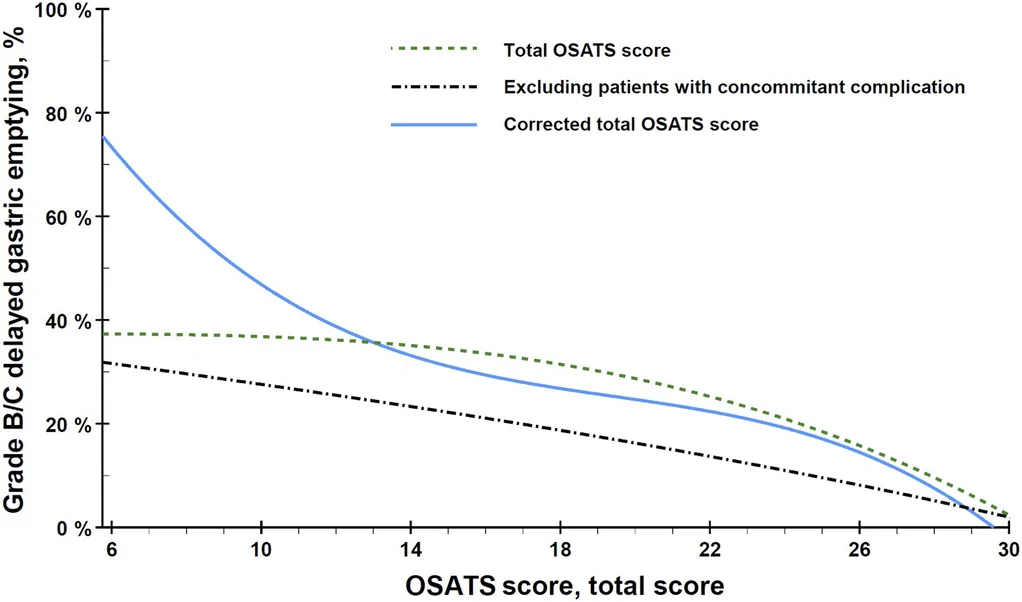
Rates of DGE along the attained total OSATS score.

OSATS score quartiles had the following ranges: Q1 = 11–17; Q2 = 18–23; Q3 = 24–26; and Q4 = 27–30. The rate of grade B/C DGE differed per OSATS quartile: Q1 = 36%, Q2 = 21%, Q3 = 28%, Q4 = 4%, *P* = 0.004. No grade B/C DGE occurred when the OSATS exceeded 28 points (19 patients), while postoperative pancreatic fistula (﻿POPF) was present in 4 patients in this group (21.1%). For details, see Figure 3. Operator receiver analysis showed significant predictive value of the total OSATS score for grade B/C DGE (AUC: 0.668, *P* < 0.001), of which the significantly predictive OSATS elements were gentleness (AUC: 0.719, *P* < 0.001), instrument handling (AUC: 0.595, *P* = 0.043), tissue exposure (AUC: 0.625, *P* = 0.009), and the summary score (AUC: 0.665, *P* < 0.001). For grade C DGE alone, the significantly predictive OSATS elements were gentleness (AUC: 0.715, *P* = 0.001) and the summary score (AUC: 0.627, *P* = 0.033). For details, see Figure 4.

An OSATS score exceeding 25 was associated with a reduced risk of grade B/C DGE (8/71, 11.3% vs 34/121, 28.1%; OR: 0.325, relative risk reduction = 59.9%, *P* = 0.006). On multivariable analysis for grade B/C DGE, OSATS ≤25 points remained a consistent predictor (OR: 2.907, *P* = 0.028, Table 3). Furthermore, OSATS showed a predictive value for grade B/C bile leakage (AUC: 0.676, *P* = 0.024), However, the RGE OSATS scores showed no predictive value for other PD complications (POPF, postoperative pancreatic hemorrhage, nor complications Clavien-Dindo grade ≥3).

**TABLE 3:** Predictive Value of OSATS for Grade B/C DGE After RPD

	Univariable		Multivariable	
Characteristic	Odds ratio	*P*	Odds ratio	*P*
Baseline				
Age, increasing years	0.123*	0.089	0.961	0.053
BMI, increasing kg/m^2^	0.049*	0.494	—	—
Sex (M)	0.780	0.473	—	—
GE comorbidity	1.016	0.966	—	—
Diabetes mellitus	1.626	0.234	—	—
Adenocarcinoma	0.611	0.279	—	—
Neoadjuvant therapy	0.620	0.403	—	—
Operative technique				
Stapled	1.162	0.669	—	—
Pylorus resection	0.170	0.003	0.195	0.040
V-loc use	4.156	<0.001	2.820	0.136
Angle type				
Type I <30 degrees to vertical	0.664	0.258	—	—
Type II ≥30 degrees to the right	1.939	0.182	—	—
Type III ≥30 degrees to the left	1.117	0.784	—	—
Cautery transection, increasing second	−0.091*	0.210	—	—
Procedures, consecutive	−0.243	<0.001	1.020	0.175
Surgeon characteristic				
University medical center	0.323	0.003	2.920	0.432
Attending surgeon	3.385	0.003	1.509	0.284
Surgical experience, increasing year	0.190*	0.008	0.967	0.530
MI surgical experience, increasing year	0.146*	0.043	0.989	0.932
Surgeon annual volume >20 PDs	0.358	0.010	0.843	0.927
Prior experience in LPD	1.104	0.794	—	—
OSATS performance cutoff				
Cutoff 23	0.473	0.084	0.574	0.204
Cutoff 24	0.348	0.016	0.432	0.062
Cutoff 25	**0.325**	**0.008**	**0.344**	**0.028**
Cutoff 26	0.106	0.002	0.067	0.004
Cutoff 27	0.106	0.030	0.104	0.048
Cutoff 28	0.079	0.052	<0.001	<0.001
Cutoff 29	0.157	0.079	<0.001	<0.001

* Values are relative to quartile 1. * = Spearman Rho.

BMI indicates body mass index; GE, gastroenterologic; LPD, laparoscopic pancreatoduodenectomy.

### Sensitivity Analysis

The adjusted OSATS score comprised of significantly predictive OSATS elements from the current study (gentleness, instrument handling, tissue exposure, and summary score), revealed a higher predictive value for DGE compared with the complete OSATS score (AUC: 0.713, *P* < 0.001 vs 0.668, *P* < 0.001, respectively). In addition, it showed a significant predictive value for grade C DGE: AUC: 0.664, *P* = 0.019 versus 0.594, *P* = 0.179, for the adjusted OSATS score versus complete OSATS score, respectively. DGE was significantly lower in the higher quartiles: Q1 = 33%; Q2 = 29%; Q3 = 20%; Q4 = 4%; *P* < 0.001. For details, see Figure 3.

Patients with DGE had significantly more concomitant postpancreatectomy hemorrhage, POPF, and bile leakage compared with patients without DGE: combined 61.9% versus 28.1%, respectively, *P* < 0.001. After excluding patients (n = 68, 35.4%) with these possible confounding factors, the predictive value of OSATS for results remained consistent (AUC: 0.671, *P* = 0.021). Moreover, in this subgroup, no DGE occurred when OSATS was above 26 points (*P* = 0.011). On multivariable analysis, including concomitant complications, OSATS ≤25 points remained a consistent risk factor (OR: 3.058, *P* = 0.030). For more details, see Appendix 2 (Supplemental Digital Content 1, http://links.lww.com/SLA/F432).

The validation grader (n = 120) showed a significant correlation with the primary grader (Rho: 0.205, *P* = 0.025). In the validity grader score, no grade B/C DGE occurred above OSATS score of 23 points (*P* = 0.139), and no grade C DGE occurred above OSATS score of 22 points (*P* = 0.067).

## DISCUSSION

This multicenter study is the first to correlate surgical performance during RGE with grade B/C DGE and a learning curve. The predictive value of OSATS for DGE remained consistent after taking variations in technique, surgeons' experience and characteristics, learning curve, and patient risk factors into account. An OSATS >25 points was associated with a 59.9% relative reduction of grade B/C DGE (8/71, 11.3% vs 34/121, 28.1%). The OSATS score over consecutive gastric anastomoses per surgeon revealed a learning curve with a turning point after 34 gastric anastomoses. Above-average scores were demonstrated after 52 RGEs.

Previously, Birkmeyer and colleagues demonstrated this effect in bariatric surgery, and more recently, OSATS scores were found to be predictive for pancreatic fistula after RPD.^18,26^ Moreover, in a simulator environment for open pancreaticojejunostomy, OSATS score had a similar cutoff value of 25/30 points, above which no POPF occurred in clinical setting vs ~50% when ≤25 points.^27^ Surprisingly, the learning curve for the gastric anastomosis (34–51 procedures) was longer than that of the pancreaticojejunostomy (25–33 procedures) and the hepaticojejunostomy (19–44 procedures) in the same cohort.^21,28^ This might be explained by the artificial organ training that was aimed at the pancreaticojejunostomy and hepaticojejunostomy.^29^ In recent reports, the learning curve of RGE was reported at 14 to 100 procedures, however, none of which reported on gastric anastomosis in robotic surgery specifically.^23^ The RGE may have received less attention as the resulting morbidity is much less than the pancreatic anastomosis.^30–32^ This study confirms again the strong link between technical performance and patient outcomes and underscores the need for enhancing bioartificial /simulated training to minimize the learning curve on patients.

Unlike the pancreaticojejunostomy and hepaticojejunostomy, it is possible to largely eliminate grade B/C DGE by reaching the highest OSATS scores (≥29, obtained in 19/192 patients, 9.9%), even while POPF was present in some of these patients. Therefore, DGE seems less subjected to biological risk factors, such as diabetes or previous comorbidity. Grade B/C DGE was also not subjected to procedure volume and surgical experience when this was corrected for OSATS score, which implies that adequate surgical performance is more important for DGE than surgeon characteristics alone. These findings emphasized the importance of training all surgeons, irrespective of their level of experience, before embarking on a minimally invasive pancreatic surgery program and inclusion of the gastric anastomosis in the artificial organ training.^13,33^ Furthermore, the DGE complications were still significantly influenced by the transection plane of the gastric remnant, that is, pylorus-resecting versus pylorus-preserving, and by concomitant complications. Consequently, after excluding patients with a POPF, bile leakage, and postpancreatectomy hemorrhage, patients with gastrojejunostomies with OSATS scores above 26 had no DGE. This implies that an OSATS performance cut-off corrected for concomitant complications is more clinically valid for DGE.

The predictive value of the total OSATS score was significant, however, not all elements of the OSATS score demonstrated a significant predictive value for grade B/C DGE. This might be reflected by the fact that quartile 3 demonstrated a higher grade B/C DGE rate than quartile 2 (28% vs 21%, respectively), as a OSATS score with only significant elements demonstrated a reduction in each quartile increment (Q1 = 38%; Q2 = 33%; Q3 = 20%; Q4 = 4%; *P* = 0.003). In addition, the overall summary score showed the lowest score compared with other OSATS elements. A possible explanation is that the graders incorporate components not represented in the OSATS score more critically in the summary score, for example, in the distance between stitches. Evidence on the type of RGE with the lowest DGE rate, for example, stapled versus sutured, is currently lacking. Future research should investigate the effect of technical factors and OSATS scores combined in the prediction of DGE. Ultimately, this could validate the assessment to determine the optimal technique for the RGE.

The current OSATS scoring system is time-consuming and not feasible for real-time assessment during surgery. During the study period, more stapling-specific OSATS scores were published (the A-OSATS-score), which might provide a better time-value balance.^34^ However, we believe that the general OSATS remains a valuable tool for surgical education and training, as it allows for structured and objective feedback on surgical performance. The time-consuming nature of OSATS evaluation does pose challenges for scalability in training. However, we believe that future developments in automated video analysis and machine learning may offer solutions to this issue. Automated systems could potentially analyze surgical videos and provide real-time feedback on technical performance, making (OSATS) assessment more efficient and scalable. In the meantime, we believe that OSATS can still be effectively used in training programs through post-operative video review and feedback sessions.

This study has some limitations. First, due to the retrospective design of this analysis, the RGE was performed in either a stapled or sutured manner. Therefore, it was not possible to correct DGE for the gastric anastomosis flow angle in multivariable analysis. Secondly, the surgical procedure was not standardized across the cohort. Intersurgeon variability occurred, where some surgeons preferred to perform the RGE at the anterior or posterior side of the stomach, while others used the transectional site of the stomach. This variability might induce bias in our results. While we corrected the most important technical factors related to DGE, the learning curves for the different approaches might be different. Furthermore, while the included surgeons per center were generally consistent, some center variability may have occurred. Third, we currently have no OSATS score for artificial organ training, therefore, the OSATS score is not validated as a performance cutoff for residents aiming to do their first clinical procedure. Finally, we acknowledge that grade C DGE can still occur after procedures with excellent technical performance, likely due to other factors. For example, anastomotic bleeds due to ulcers. In our cohort, some surgeons mentioned the occurrence of anastomotic intraluminal bleeds resulting in grade C DGE, these bleeds were found and cured with endoscopic intervention. We had no information on the specific endoscopic interventions.

## CONCLUSIONS

Using OSATS to score surgical performance in gastrojejunostomy in RPD is a useful tool for identifying a clinically relevant learning curve. Furthermore, OSATS was an independent predictor for the rate of grade B/C DGE. Finally, OSATS could serve as a training tool for assessment of high quality RGE, the OSATS-based performance bar requires further research.

## Supplementary Material

## References

[1] Pędziwiatr M Małczak P Pisarska M . Minimally invasive versus open pancreatoduodenectomy-systematic review and meta-analysis. Langenbecks Arch Surg. 2017;402:841–851. 28488004 10.1007/s00423-017-1583-8 PMC5506213

[2] van Hilst J de Rooij T Abu Hilal M . Worldwide survey on opinions and use of minimally invasive pancreatic resection. HPB (Oxford). 2017;19:190–204. 28215904 10.1016/j.hpb.2017.01.011

[3] Klotz R Mihaljevic AL Kulu Y . Robotic versus open partial pancreatoduodenectomy (EUROPA): a randomised controlled stage 2b trial. Lancet Reg Health Eur. 2024;39:100864. 38420108 10.1016/j.lanepe.2024.100864 PMC10899052

[4] Zureikat AH Postlewait LM Liu Y . A multi-institutional comparison of perioperative outcomes of robotic and open pancreaticoduodenectomy. Ann Surg. 2016;264:640–649. 27433907 10.1097/SLA.0000000000001869

[5] Giulianotti PC Sbrana F Bianco FM . Robot-assisted laparoscopic pancreatic surgery: single-surgeon experience. Surg Endosc. 2010;24:1646–1657. 20063016 10.1007/s00464-009-0825-4

[6] Boone BA Zenati M Hogg ME . Assessment of quality outcomes for robotic pancreaticoduodenectomy: identification of the learning curve. JAMA Surg. 2015;150:416–422. 25761143 10.1001/jamasurg.2015.17

[7] Peng L Lin S Li Y . Systematic review and meta-analysis of robotic versus open pancreaticoduodenectomy. Surg Endosc. 2017;31:3085–3097. 27928665 10.1007/s00464-016-5371-2

[8] de Rooij T Lu MZ Steen MW . Minimally invasive versus open pancreatoduodenectomy: systematic review and meta-analysis of comparative cohort and registry studies 2016 Ann Surg, 264:257–267. 26863398 10.1097/SLA.0000000000001660

[9] Bao PQ Mazirka PO Watkins KT . Retrospective comparison of robot-assisted minimally invasive versus open pancreaticoduodenectomy for periampullary neoplasms. J Gastrointest Surg. 2014;18:682–689. 24234245 10.1007/s11605-013-2410-3

[10] Chen S Chen JZ Zhan Q . Robot-assisted laparoscopic versus open pancreaticoduodenectomy: a prospective, matched, mid-term follow-up study. Surg Endosc. 2015;29:3698–3711. 25761559 10.1007/s00464-015-4140-y

[11] Jung JP Zenati MS Dhir M . Use of video review to investigate technical factors that may be associated with delayed gastric emptying after pancreaticoduodenectomy. JAMA Surg. 2018;153:918–927. 29998288 10.1001/jamasurg.2018.2089 PMC6584315

[12] Kamarajah SK Bundred JR Alessandri G . A systematic review and network-meta-analysis of gastro-enteric reconstruction techniques following pancreatoduodenectomy to reduce delayed gastric emptying. World J Surg. 2020;44:2314–2322. 32166469 10.1007/s00268-020-05459-5

[13] Zwart MJW Nota CLM de Rooij T . Outcomes of a multicenter training program in robotic pancreatoduodenectomy (LAELAPS-3). Ann Surg. 2022;276:e886–e895. 33534227 10.1097/SLA.0000000000004783

[14] Malleo G Vollmer CM Jr . Postpancreatectomy complications and management. Surg Clin North Am. 2016;96:1313–1336. 27865280 10.1016/j.suc.2016.07.013

[15] Emmen AMLH Zwart MJW Khatkov IE . Robot-assisted versus laparoscopic pancreatoduodenectomy: a pan-European multicenter propensity-matched study. Surgery. 2024;175:1587–1594. 38570225 10.1016/j.surg.2024.02.015

[16] Wang M Li D Chen R . Laparoscopic versus open pancreatoduodenectomy for pancreatic or periampullary tumours: a multicentre, open-label, randomised controlled trial. Lancet Gastroenterol Hepatol. 2021;6:438–447. 33915091 10.1016/S2468-1253(21)00054-6

[17] Eisenberg JD Rosato EL Lavu H . Delayed gastric emptying after pancreaticoduodenectomy: an analysis of risk factors and cost. J Gastrointest Surg. 2015;19:1572–1580. 26170145 10.1007/s11605-015-2865-5

[18] Hogg ME Zenati M Novak S . Grading of surgeon technical performance predicts postoperative pancreatic fistula for pancreaticoduodenectomy independent of patient-related variables. Ann Surg. 2016;264:482–491. 27433897 10.1097/SLA.0000000000001862

[19] Birkmeyer JD Finks JF O'Reilly A . Surgical skill and complication rates after bariatric surgery. N Engl J Med. 2013;369:1434–1442. 24106936 10.1056/NEJMsa1300625

[20] Martin JA Regehr G Reznick R . Objective structured assessment of technical skill (OSATS) for surgical residents. Br J Surg. 1997;84:273–278. 9052454 10.1046/j.1365-2168.1997.02502.x

[21] van den Broek BLJ Zwart MJW Bonsing BA . Video grading of pancreatic anastomoses during robotic pancreatoduodenectomy to assess both learning curve and the risk of pancreatic fistula - a post hoc analysis of the LAELAPS-3 training program. Ann Surg. 2023;278:e1048–e1054. 36727842 10.1097/SLA.0000000000005796 PMC10549894

[22] Kendrick ML van Hilst J Boggi U . Minimally invasive pancreatoduodenectomy. HPB (Oxford). 2017;19:215–224. 28317658 10.1016/j.hpb.2017.01.023

[23] Müller PC Kuemmerli C Cizmic A . Learning curves in open, laparoscopic, and robotic pancreatic surgery: a systematic review and proposal of a standardization. Ann Surg Open. 2022;3:e111. 37600094 10.1097/AS9.0000000000000111 PMC10431463

[24] Masui T Kubora T Nakanishi Y . The flow angle beneath the gastrojejunostomy predicts delayed gastric emptying in Roux-en-Y reconstruction after distal gastrectomy. Gastric Cancer. 2012;15:281–286. 22041869 10.1007/s10120-011-0107-4

[25] Sullivan GM Artino AR Jr . Analyzing and interpreting data from Likert-type scales. J Grad Med Educ. 2013;5:541–542. 24454995 10.4300/JGME-5-4-18 PMC3886444

[26] Vaidya A Aydin A Ridgley J . Current status of technical skills assessment tools in surgery: a systematic review. J Surg Res. 2020;246:342–378. 31690531 10.1016/j.jss.2019.09.006

[27] Mizunuma K Kurashima Y Poudel S . Surgical skills assessment of pancreaticojejunostomy using a simulator may predict patient outcomes: a multicenter prospective observational study. Surgery. 2023;173:1374–1380. 37003952 10.1016/j.surg.2023.02.027

[28] Zwart M van den Broek B Geraedts T . Video analysis of hepaticojejunostomy to predict biliary complications after robotic pancreatoduodenectomy. Pancreatology. 2022;22:e88–e89.

[29] Nota CL Zwart MJ Fong Y . Developing a robotic pancreas program: the Dutch experience. J Vis Surg. 2017;3:106. 29078666 10.21037/jovs.2017.07.02 PMC5638300

[30] Kauffels A Reichert M Askevold I . Establishing robotic bariatric surgery at an academic tertiary hospital: a learning curve analysis for totally robotic Roux-en-Y gastric bypass. J Robot Surg. 2023;17:577–585. 35994194 10.1007/s11701-022-01454-1 PMC10076403

[31] Buchs NC Pugin F Bucher P . Learning curve for robot-assisted Roux-en-Y gastric bypass. Surg Endosc. 2012;26:1116–1121. 22044973 10.1007/s00464-011-2008-3

[32] Bindal V Gonzalez-Heredia R Masrur M . Technique evolution, learning curve, and outcomes of 200 robot-assisted gastric bypass procedures: a 5-year experience. Obes Surg. 2015;25:997–1002. 25394589 10.1007/s11695-014-1502-9

[33] Zwart MJW van den Broek B de Graaf N . The feasibility, proficiency, and mastery learning curves in 635 robotic pancreatoduodenectomies following a multicenter training program: standing on the shoulders of giants. Ann Surg. 2023;6:e1232–e1241. 10.1097/SLA.0000000000005928 PMC10631507 37288547

[34] Schmidt MW Haney CM Kowalewski KF . Development and validity evidence of an objective structured assessment of technical skills score for minimally invasive linear-stapled, hand-sewn intestinal anastomoses: the A-OSATS score. Surg Endosc. 2022;36:4529–4541. 34755235 10.1007/s00464-021-08806-2 PMC9085690

