# Supplementary material for: Video Assessment of Surgical Performance in Robotic Gastroenterostomy During Pancreatoduodenectomy: Evaluating the Learning Curve and Risk of Delayed Gastric Emptying
Source: Ann Surg. 2025 Mar 12;284(4):787–95. doi: 10.1097/SLA.0000000000006692 (PMC13557470; doi:10.1097/SLA.0000000000006692)
Supplement: Supplementary file 1 [file sla-284-787-s001.docx]

# APPENDIX

## Appendix 1. Flow angle figure.

Classification of gastrojejunostomy according to Masui *et al*.,^24^, and modified by Jung *et al.*^11^ Type I gastrojejunostomy direction being equal or less than 30° to vertical (the vertebral line), type II gastrojejunostomy direction angles more than 30° to the right side, type III gastrojejunostomy direction being more than 30° to the left (spleen) side.

## Appendix 2. Supplementary table 1.

| **OSATS score performance cut-off including variable other complications (POPF, BL, PPH)** | | | | |  |
| --- | --- | --- | --- | --- | --- |
|  | **Univariable** | | **Multivariable** | | |
| **Characteristic** | **Odds ratio** | ***P* value** | **Odds ratio** | ***P* value** | |
| Cut-off 22 | 0.546 | 0.268 | 0.528 | 0.172 | |
| Cut-off 23 | 0.481 | 0.194 | 0.519 | 0.162 | |
| Cut-off 24 | 0.306 | 0.065 | 0.400 | 0.057 | |
| **Cut-off 25** | **0.371** | **0.128** | **0.327** | **0.030** | |
| Cut-off 26 | 0.008 | 0.011 | 0.069 | 0.005 | |
| Cut-off 27 | 0.173 | 0.076 | 0.119 | 0.062 | |
| Cut-off 28 | 0.248 | 0.140 | * | * | |
| Cut-off 29 | 0.406 | 0.292 | * | * | |
| *<0.001. | | | | |  |
